# Longitudinal fecal hormone monitoring of adrenocortical function in zoo housed fishing cats (*Prionailurus viverrinus*) during institutional transfers and breeding introductions

**DOI:** 10.1371/journal.pone.0230239

**Published:** 2020-03-18

**Authors:** Jilian M. Fazio, Elizabeth W. Freeman, Erika Bauer, Larry Rockwood, Janine L. Brown, Katharine Hope, Jessica Siegal-Willott, E. C. M. Parsons

**Affiliations:** 1 Center for Animal Care Sciences, Smithsonian’s National Zoological Park, Washington, District of Columbia, United States of America; 2 Department of Environmental Science and Policy, George Mason University, Fairfax, Virginia, United States of America; 3 School of Integrative Studies, George Mason University, Fairfax, Virginia, United States of America; 4 Department of Biology, George Mason University, Fairfax, Virginia, United States of America; 5 Center for Species Survival, Smithsonian’s Conservation Biology Institute, Front Royal, Virginia, United States of America; Centre for Cellular and Molecular Biology, INDIA

## Abstract

The *ex situ* population of fishing cats (*Prionailurus viverrinus*) has become increasingly important for the conservation of this species. Unfortunately, captivity-induced stress is a concern and potential factor for lack of breeding success in this small felid, resulting in an unsustainable population. The objectives of this study were to: 1) validate an enzyme immunoassay for monitoring of fecal glucocorticoid metabolite (FGM) concentrations in the fishing cat; 2) identify potential exogenous stressors in the captive environment; 3) pinpoint management techniques that may lower FGM concentrations; and 4) determine if FGM concentrations are related to breeding success. Through a successful adrenocorticotrophic hormone challenge and additional laboratory methods, a cortisol enzyme immunoassay was validated as an effective tool for detecting FGM in this species. Between 2010 and 2013, longitudinal FGM monitoring was conducted in 26 fishing cats in the North American Species Survival Plan^®^. Exogenous stressors that elevated FGM concentrations included: chemical immobilizations; permanent transfers between facilities; construction; facility events; and fights/aggression among breeding pairs. Management factors that lowered FGM concentrations included: increased animal-keeper interaction through formal training; and providing indoor, off-exhibit, holding areas. In addition, social housing of individuals (either established breeding pairs or same sex pairs) decreased FGM concentrations. Individuals with breeding success (defined as observed copulations during the study period) also had lower FGM concentrations than unsuccessful individuals. Findings indicate that management factors play a role in lowering glucocorticoid (stress) levels in fishing cats, which may ultimately affect breeding success in the *ex situ* population.

## Introduction

The fishing cat (*Prionailurus viverrinus*) is classified as “vulnerable” by the International Union for the Conservation of Nature (IUCN) mainly due to a rapid decline in wild populations over the past two decades [1]. Thus, the *ex situ* population has become increasingly important for the conservation of the species [2]. The current captive population, however, is unsustainable due to low founder numbers, high mean kinship, and low reproductive success [2,3]. Although managing fishing cats in a zoological setting is necessary for their conservation, little is known about how members of this species vary in their sensitivity and stress response to environmental changes and different management strategies in a zoos.

In felids, stressful events are known to lead to infanticide by females and attacks by males during breeding introductions, which cause injury or death [4]. Studbook data from 1985–2011 showed high rates of infant mortality in the fishing cat: 45% in male and 36% in female kittens [5]. Management strategies, such as hand-rearing, can also affect stress levels and social behaviors in felids. In hand-reared clouded leopards (*Neofelis nebulosa*), individuals have actually been found to have lower levels of fecal glucocorticoid metabolite (FGM) than mother-reared individuals [6]. Little is known about how to effectively manage fishing cats in zoos, so determining strategies that mitigate stress could increase mate compatibility and decrease infant mortality.

Stressful events can be measured as fluctuations in adrenal hormones—specifically glucocorticoids—that are regulated by the hypothalamus-pituitary-adrenal (HPA) axis. Noninvasive monitoring of fecal glucocorticoid metabolites (FGM) is one effective way of assessing adrenal activity in captive felid species [7–10]. Increased glucocorticoids have been shown to have a direct effect on reproduction in mammals by interrupting the normal function of the hypothalamus-pituitary-gonadal (HPG) axis [11–13]. Small felids in particular are known to be extremely sensitive to environmental stressors as they are often not only a predator, but also a prey species in their natural habitat [8,14]. Depending on the species, felid management is improved through offering of hiding spaces [14], increased enclosure height [6], and ample space to retreat from stressors [4]. These factors can all influence the degree of environmental stress on an individual and ultimately affect their welfare and reproductive success.

Both enzyme-based (EIA) and radioactive-based (RIA) immunoassays have been used to monitor FGM’s in felids. Because inter-species variation in FGM concentrations and patterns exists, both physiological and biological validations are needed for any new immunoassay procedure [6,9,15]. An adrenocorticotrophic hormone (ACTH) challenge is the best way to demonstrate the physiological validity of an assay, by showing that increases in endogenous glucocorticoids in response to ACTH are reflected in excretia. Glucocorticoid responses to ACTH also can also help determine variation in excretory patterns due to sex, age or species-specific metabolism [16]. ACTH challenges have been conducted on several felid species to validate immunoassay procedures, including the cheetah [9,17,18], clouded leopard [6], ocelot (*Leopardus pardalis*) [19], oncilla (*Leopardus tigrinus*) and margay (*Leoparadus wiedi*) [20], jaguar (*Panthera onca*) [15], canadian lynx (*Lynx canadensis*) [21] and fishing cat [7].

The primary objectives of this study were to validate an immunoassay for use in fishing cats in order to monitor adrenal function longitudinally during institutional transfers and breeding introductions. It was hypothesized that there would be measurable differences in FGM concentrations due to exogenous stressors and management factors, and that breeding success would be affected by levels of FGM.

## Materials and methods

### Animal subjects and sample collection

Animal subjects (Table 1) were fishing cats recommended for institutional transfer or breeding by the Fishing Cat Species Survival Program (SSP) between 2010 and 2013. These included 13 male and 13 female fishing cats, which comprised 15 unique breeding pairs and incorporated 20 institutional transfers at 16 facilities accredited by the Association of Zoos and Aquariums (AZA). All facilities followed housing, diet and management guidelines set out by the AZA (detailed in [22]). This study was authorized by Smithsonian’s National Zoological Park’s (SNZP) Animal Care and Use Committee (SNZP-ACUC #10–26 and #14–13) and was approved by individual facilities’ Institutional Animal Care and Use committees, when necessary.

**Table 1:** Animal subjects. Studbook numbers (SB #) were used to identify individuals and mates were based on Species Survival Program recommendations. Age was determined at the start of the study for each cat. Rearing is listed as either mother—raised by the dam or hand—raised by humans at any time prior to weaning. Breeding success was defined as copulations observed (Y) or no copulations observed (N). All individuals were sexually mature at the time of the study.

Fishing Cat SB#	Sex	Age	Rearing	Study year	Intended mate	Breeding success
440	M	12	Mother	2010	722	N
		13		2011	732	N
497	M	8	Hand	2010	694	N
542	F	8	Hand	2011	661*	Y
633	F	6	Mother	2010	781	Y
		7		2011	781	Y
		8		2012	781	Y
651	F	7	Hand	2010	721	N
652	F	7	Hand	2010	780	Y
663	M	6	Hand	2010	733	N
		9		2012	776	N
657	S	9	Mother	2011	-	-
664	F	8	Hand	2011	778	Y
687	F	5	Mother	2010	759	Y
		7		2012	-	-
694	F	5	Hand	2010	497	N
		7		2012	721	N
720	M	4	Mother	2010	-	-
		5		2011	-	-
721	M	4	Mother	2010	651	N
		6		2012	694	N
722	F	4	Mother	2010	440	N
		5		2011	-	-
732	F	4	Mother	2011	440	N
733	F	3	Mother	2010	663	N
		5		2012	759	N
759	M	3	Mother	2010	687	Y
		6		2012	733	N
776	F	4	Mother	2012	663	N
777	M	3	Hand	2010	-	-
778	M	4	Mother	2011	664	Y
779	M	1	Mother	2010	776	N
		3		2012	733	N
780	M	1	Mother	2010	652	Y
781	M	1	Mother	2010	633	Y
		2		2011	633	Y
		3		2012	633	Y
950	F	1	Mother	2011	-	-
		2		2012	779	N
959	M	1	Mother	2012	-	-
960	M	1	Mother	2012	-	-

*SB# 661 was an intended mate, however no fecal samples were collected on this individual so he is only listed as a reference but not included as an individual in the study analysis.

Fecal samples were collected every day for 30 days prior to transfer and throughout quarantine. Once a cat was released from quarantine, every other day fecal collections began for up to 1 year on all cats, transferred or stationary, and housed at the receiving institutions. Fecal samples were also collected every other day for 1 year from fishing cats recommended for breeding, but not transfer. All fecal samples were collected within 24 hours of being voided and stored frozen at -20°C. In some instances, fecal markers were used to identify individual fecal samples in cats that were socially housed. One of two types of markers were added to the individual’s daily diet: food dye (#601, Wilton icing color, Wilton Industries, Woodridge, IL, USA); or various colors of non-toxic glitter (Glitterex Corporation, Cranford, NJ, USA). SB# 661 was an intended mate; however, no fecal samples were collected, so he is included as a reference only, and not included in further evaluations.

### ACTH challenge

An ACTH challenge was performed on one adult female fishing cat (SB# 633) held at the National Zoo, Washington DC, to determine the timing of FGM release and provide physiological validation for the EIA used in the study. A single intramuscular injection of 300 IU (3.75 ml) synthetic ACTH gel (Corticotrophin LA 80 IU/ml; Wedgewood Pharmacy, Swedesboro, NJ, USA) was given at 10:00 am on September 27, 2014, to simulate the natural release of ACTH. The cat (8.1 kg) was netted and restrained for injection in the left lateral thigh. All fecal samples produced were collected for 5 days (n = 5) before and 3 days after injection (n = 4), and then one sample/day was collected for another 2 days (n = 2). All samples were labeled with the time and date of excretion and stored at– 20°C in plastic bags until analysis. ACTH challenge samples were analyzed using four immunoassays (Table 2) to identify the most appropriate assay to measure FGM in this species.

**Table 2:** Immunoassays used to evaluate fecal samples from an adrenocorticotrophic hormone challenge (ACTH). Results indicated a high correlation for both validation methods used; parallel displacement curves and recovery of the standards. Cross-reactivities of each assay with associated glucocorticoid metabolites are indicated as well as the optimal dilution used for samples on each assay.

Assay	Product Information	Parallelism	Recovery		Cross-reactivities	Dilution
		Correlation coefficient	Equation	Correlation coefficient		
Cortisol EIA (single antibody)	R4866 supplied by C.J. Munro, University of California, Davis, CA, USA	r = 0.98	y = 0.88x + 89.17	r = 0.98	cortisol at 100%, prednisolone (9.9%), prednisone (6.3%), cortisone (5%), other metabolites at <1%	1:200
Cortisol RIA (double antibody)	Coat-A-Count, PITKCO-9, 2010-10-21, Siemens, Los Angeles, CA, USA	r = 0.98	y = 1.40x +0.56	r = 0.99	prednisolone (76%), 11-deoxycortisol (11.4%), methylprednisone (11%), prednisone (2.3%), betamethasone (1.6%), other metabolites at <1%	1:8
Corticosterone EIA (double antibody)	R0006 supplied by C.J. Munro, UC Davis, Davis, CA, USA	r = 0.96	y = 0.82x + 15.27	r = 0.99	corticosterone 100%, deoxycorticosterone 14.25%, progesterone 2.65%, other metabolites at <1%	1:64
Corticosterone RIA (double antibody)	I^125^ kit ImmuChem, MP Biomedicals, LLC Diagnostics Division Orangeburg, NY, USA	r = 0.95	y = 1.31x + 51.92	r = 0.99	corticosterone 100%, other metabolites at <1%	1:200

### Noninvasive fecal hormone analysis

Initial fecal preparation involved drying samples in a lyophilizer (VirTis Ultra 35XL, SP Scientific, Warminster, PA) for 5 days [23] Dried samples were then pulverized with a mallet to produce a fine fecal powder. Hormones were extracted by adding 0.20 ± 0.02 g of dried/crushed fecal powder to 5mL of 90% ethanol (EtOH) into 16x125 mm glass tubes (Fisherbrand, Thermo Fisher, Pittsburgh, PA, USA). This mixture was then subjected to a 30-minute modified shaking technique, based upon established protocols [16], using a 2.5 amp, 120 volt, large capacity mixer set at motor speed 60 (Glas-col, Terre Haute, IN, USA). The samples were then centrifuged at 2000 rpm for 20 minutes and the supernatant poured off. The diluent was reconstituted with 5 ml 90% EtOH, vortexed (pulse rate 1/s, speed 65; Multi-tube Vortexer; Glas-Col, Terre Haute, IN, USA) and shaken for 30 seconds before it was again centrifuged at 2000 rpm for 15 minutes. The supernatant was combined with the original supernatant for each sample and dried under forced hot air overnight. The sample was then brought up in 1 ml 100% methanol (MeOH) and redried. Finally, the ‘neat’ solution was created by adding 1 ml of preservative-free phosphate buffer (0.2 M NaH_2_PO_4_, 0.2 M Na_2_HPO_4_, 0.15 M NaCl; pH 7.0) to the sample; all sample dilutions (1:100–1:500) were created from the neat sample using the same buffer. Samples were analyzed in duplicate and results were expressed as μg/g of dry feces.

### Assay validation

Standard procedures were used to validate the four hormone assays [9,16,23]. All serial dilutions of fecal extracts (neat to 1:512) demonstrated parallelism with slopes matching the standard curve at r = > 0.95. Mass recovery tests were performed by adding known but varying concentrations of exogenous cortisol or corticosterone (standards) to equal volumes of diluted fecal extract and calculating the difference between the two. All recoveries indicated no interference in antibody-antigen binding due to the presence of substances within the fecal matrix. Extraction efficiencies for cortisol were monitored using a previously validated radioactive tracer (20,000 dpm ^3^H-cortisol; Perkin Elmer, Boston, MA, USA). Dry fecal samples (0.2 g) were spiked with 100 μl of tracer prior to extraction. Only extraction efficiencies above 60% were used to ensure a high degree of consistency within sample extraction. The mean extraction efficiency of all fecal samples was 78.80% ± 0.01% (n = 6801).

Intra-assay quality control was maintained during longitudinal monitoring by re-running samples with a duplicate coefficient of variation (CV) of > 10%. Inter-assay control monitors were maintained for standards, and all assays falling outside of ± 2 SD were re-done. Two internal controls analyzed on each plate had inter-assay CVs of 10.6% and 7.0% for high and low controls, respectively (n = 348).

The peak fecal sample obtained during the ACTH challenge was used for reverse-phase high performance liquid chromatography (HPLC; Varian ProStar; Varian Analytical Instruments, Lexington, MA, USA) to identify steroid hormone metabolites in fishing cat fecal extracts [24]. The methanol extract was purified on a solid-phase C-18 matrix cartridge (#01–10, Spice Cartridge, Analtech Inc., Newark, DE, USA) and dried under forced air. This extract was reconstituted in 0.3 ml MeOH, sonicated for 5 min, and 0.05 ml of the resulting solution was loaded onto a reverse phase C18 HPLC column (Agilent Technologies, Santa Clara, CA, USA). One milliliter fractions were collected using a 20–80% gradient of methanol:water over an 80 min period (1 ml/min flow rate). After separation, an aliquot of each fraction was counted on a multi-purpose β-radiation scintillation counter (LS 6500, Beckman Coulter, Brea, CA, USA), and the remainder of each fraction was dried and reconstituted in 0.25 ml preservative-free phosphate buffer. Immunoreactivity of each fraction was quantified using a cortisol EIA.

### Longitudinal monitoring and exogenous stressors

A Primary Keeper Questionnaire, described in Fazio et al. 2018 [25], was filled out once for each cat, within each study year, to obtain management variables during that study period. This information was obtained for transferred cats at both the sending and receiving institution. Animal care staff also recorded dates of potential exogenous stressors during the study period. These included, but were not limited to: transfers within and between institutions; physical restraints; sedation for medical procedures; hospitalizations; chronic illness; exhibit and management changes; and construction. Information was classified and recoded as Events—potential exogenous stressors (Table 3). Due to sporadic sample collection and the fact that many of these stressors were longer in duration (i.e., construction, internal moves), all hormone samples 1 week post Event were coded with the appropriate potential stressor. If multiple events happened in the same time frame, the event with the longest impact was prioritized (i.e., institutional transfer trumped a chemical immobilizations (sedation exam). These acute changes in adrenocortical activity were evaluated over time, throughout the study period.

**Table 3:** Definitions and groupings for potential exogenous stressors (Events) and classification for housing (Social status) of fishing cats.

**Events**	
Internal transfer	Cat moves to a new enclosure within the same facility. Housing, staff and management all may vary
New area introduction	No olfactory stimulation of other fishing cats—primarily coded for unpaired individuals
Sedation exam	Cat was anesthetized
Exam no sedation	Dart, pole inject, netting, crating
Illness	Medication administered for vomit, blood in stool, loose stool or eye discharge
Hospital stay for illness	Cat moved to hospital for >24 hours for care due to an illness
Major exhibit change	Within existing exhibit, new large items introduced or excluded from areas
Major management change	Shift in time they are rotated through exhibit
Other events	Holiday or facility event, construction, new or unusual enrichment
Copulation	Observed mounting or attempted mounting
Fights/aggression	Stalking/displacing cat during intro or an actual fight
**Social status**	**Definition**
Individually housed	Cat is housed on their own with no visual or olfactory stimulation of another fishing cat
Visual no howdy	Cat is housed alone, but has visual access to another fishing cat
Howdy introduction	Cat is housed alone, but has visual and partial physical (through mesh) contact to another fishing cat
Physical introduction	Two fishing cats are physically placed together for less than 12 hours
Rotating the same areas with pair	Two fishing cats are sharing the same area (exhibit or holding space, but are not physically together)
Housed 24 hours with pair	Breeding pair is housed together 24 hours a day
Housed 12 hours with pair	Breeding pair is housed together 12 hours a day
Separated from pair	An existing breeding pair is separated due to shipment, illness, parturition etc.
Same sex pair	A non-breeding same sex pair of fishing cats housed together
Dam and kittens	A female housed with offspring

### Breeding pairs

Facilities with breeding pairs also filled out a Breeding Introduction Record, described in Fazio et al. 2018 [25] to document when fishing cats recommended for breeding were introduced during the study period. Survey data were used to determine Social Status—how the individual was housed during the study period (Table 3). Social status was recorded continuously throughout the study for comparison with FGM concentrations.

### Hormone analysis

R package version 1.0 (Longitudinal Analysis of Hormone Data, [26]) was used to obtain FGM measures for each individual cat by study year. Baseline values were obtained using an iterative process [23], which involved systematically eliminating samples that were higher than + 2.5 standard deviation (SD) from the mean [19,27]. Significant peaks were then considered to be any samples above 2.5 SD of the mean baseline. A higher SD was chosen in order to account for any slight seasonal effects that may be seen in this species [7] as animal subjects were housed throughout the United States with variable climate and different exposure to the outdoors. Baseline measures were calculated for two separate time periods during each study year for each cat, including: 1) Preship—transferred cats = the month prior to shipment; stationary (non-transferred cats) = the month prior to the transferred cat’s release from quarantine; 2) Post-release—after quarantine release, for up to one year, for both transferred and stationary cats. Independent study year values were used for analysis of management variables and breeding behaviors.

For cats monitored during severe illness (n = 3), Student’s t-test (two-tailed), using Levene’s test for equal variance, was used to determine if there were significant differences from baseline during confirmed periods of illness. If a significant difference was found (P < 0.05) during periods of illness versus periods of health, all of the values associated with the illness were then excluded from the analyses detailed below, but were used to inform the discussion of the effects of severe physiological stressors on these individuals.

The return to baseline, or adjustment period for the cat to settle in after transfer, was determined for each transferred cat by identifying the first date that represented a full month of samples with ≤1 significant peak above baseline (e.g. >2.5 SD). Results from each individual were averaged to estimate the number of days to return to baseline after institutional transfer in fishing cats. In addition, monthly averages for mean FGM concentrations were obtained for each month of transfer, including quarantine, through 12 months post-release from quarantine, for both transferred and stationary cats. A regression analysis was used to determine the potential effect of time on FGM measures.

### Data analysis

To assess the potential effects of management on FGM, a Generalized Linear Mixed Model (GLMM) was conducted on pre-ship and post-release baseline concentration (calculated in hormLong as described above) for each cat, at each institution in which they were housed (for example pre-ship values for sending institution and post-release values for receiving institution for cats that were transferred were both used in order to compare to variables at those locations). A GLMM was well suited for these data as it accounts for repeated measures of the same individuals and minimizes risk of pseudoreplication by taking individual cats into account as a random effect. Fixed effects included: transfer (Yes/No); age; total space available to the animal (m^2^); number of indoor off exhibit enclosures; days/month trained (0–30); days/month receiving behavioral enrichment (0–30); number of keepers. Two additional variables were also included. The first was how busy was the holding area (where the cats were brought while keepers accessed their exhibit area for cleaning and maintenance); coded as: 1 –quiet (1–2 people once or twice a day), 2 –moderately active (1–2 people several times a day, 3 –active (several people in and out but all primary keepers), 4 –moderately busy (Several people in and out including strangers), 5 –busy (multitudes of people in and out). The second variable was how often the cats had access outside; coded as: 1–24 hours, 2 –day only, 3 –night only, 4 –varied, 5 –none (indicating an individual housed indoors only). Off exhibit was defined as an area away from public viewing. These management variables were selected after running a Pearson’s correlation and eliminating variables that were highly correlated (P < 0.05) to avoid collinearity within the model (S1 Table).

Three separate GLMM models were run on raw FGM concentrations to examine concentration as a dependent variable with the two variables Events and Social Housing. For these models, individual was as a random effect, and included: 1) sex, rearing, age, and transfer as fixed effects; 2) sex, rearing, age, transfer and Events (Table 3) as fixed effects; and 3) sex, rearing, age, transfers, and Social Housing (Table 3) as fixed effects.

A GLMM, with a binomial distribution and logit link function, was used to test if any of five different post-release FGM measures could predict reproductive success. These included: 1) Mean; 2) Baseline; 3) Cutoff—the value of 2.5 standard deviations above baseline; 4) Peak mean—an average of the ‘peak’ values (those greater than 2.5 SD); and 5) Proportion of peaks—total number of peaks within a given time period/total number of samples within that time period.

The GLMM analysis was conducted only on fishing cats intended as breeding pairs, and thus undergoing breeding introductions. Individuals were classified as either successful (those with observed copulations reported during the study period) or unsuccessful (no observed copulations). Individual cat was included as a random effect with age, rearing and transfers as fixed effects. Each FGM measure (mean, baseline, cutoff, peak mean, and proportion of peaks) calculated for the post-release period only, was entered as a fixed effect separately to determine which of the five could predict success. Statistical analyses were performed primarily in SPSS v22 (IBM, Somers, N.Y., U.S.A.). Tests for normality were run using a Kolmogorov-Smirnov Z test. All significant results (P < 0.05) are reported as Mean ± Standard Error (SE).

## Results

### ACTH challenge

**Fig 1:**
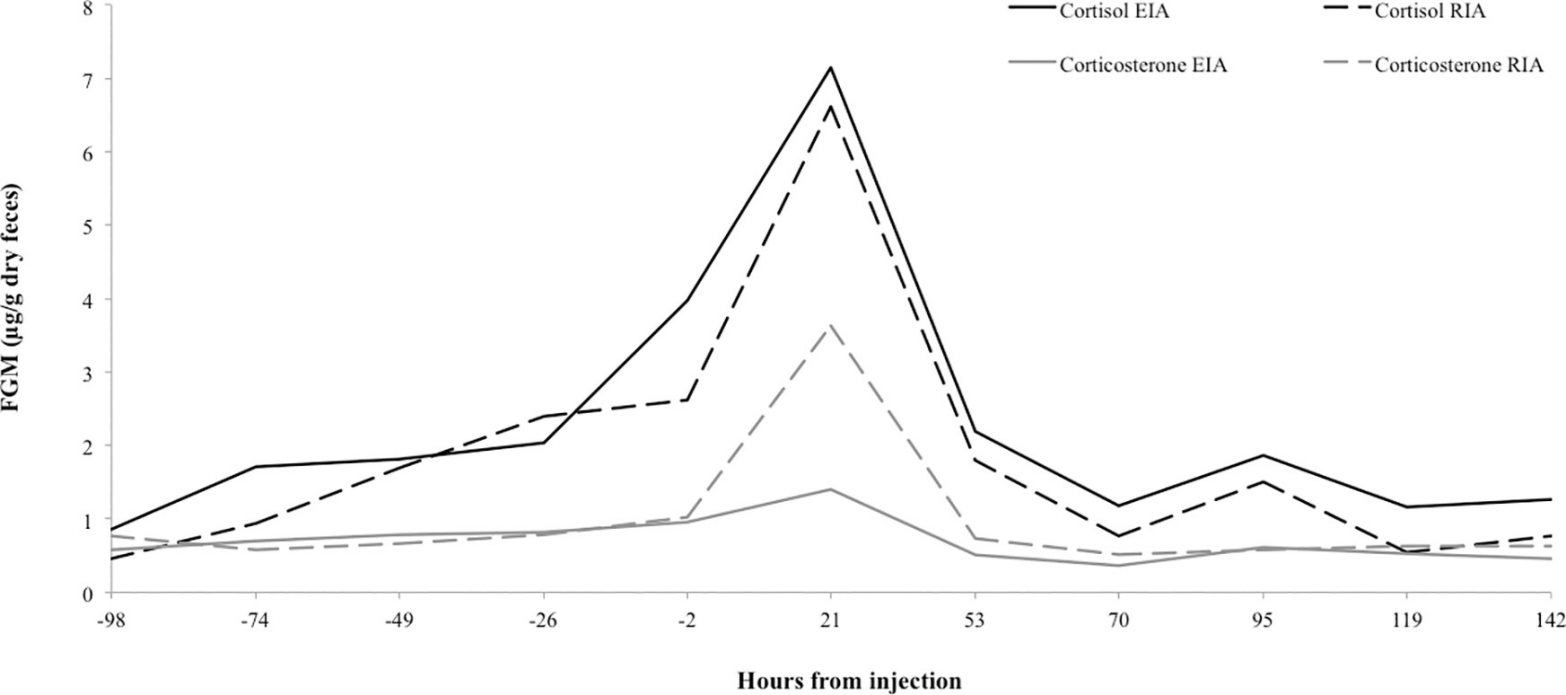
Comparison of four immunoassays employed to characterize fecal glucocorticoid metabolites (FGM) in the feces of a single female fishing cat (SB# 633) during an adrenocorticotrophic challenge. The cortisol RIA (Coat-A-Count, PITKCO-9, 2010-10-21, Siemens, Los Angeles, CA, USA) and corticosterone RIA (I^125^ kit ImmuChem, MP Biomedicals, LLC Diagnostics Division Orangeburg, NY, USA) also had measurable peaks above their pretreatment means (not shown here). The corticosterone EIA (R0006 supplied by C.J. Munro) did not.

A single peak was observed at 21 hours post injection in all four glucocorticoid assays (Fig 1). The cortisol EIA (R4866) showed a peak that exceeded baseline by 344% of the pretreatment mean (mean = average of pretreatment samples and considered 100%) [9]. FGM concentrations returned to baseline by 48 hours post-injection and remained there for the monitoring period (141 hours post-injection). Post-ACTH FGM peak concentrations when compared to the pretreatment mean for that particular immunoassays were: 408% for the cortisol RIA; 194% for the corticosterone EIA; and 377% for the corticosterone RIA. Given these results, all assays measured a significant increase in glucocorticoids post ACTH; however, the cortisol EIA was selected as it was the most cost effective.

### High performance liquid chromatography

Analysis of the high performance liquid chromatography (HPLC) -separated fishing cat fecal eluates revealed the presence of two glucocorticoid metabolites associated with the ^3^H-cortisol (fractions 39–41) and ^3^H-corticosterone (fractions 45–47) tracers. However, only approximately 4% of the immunoreactivity (quantified by the cortisol EIA) was associated with these peaks. A majority of eluted polar peaks occurred at fractions 4–10 indicating a large amount of nonenzyme-hydrolyzable, water-soluble metabolites that accounted for 96% of total immunoreactivity (S1 Fig).

### Management factors

The GLMM of management factors showed that the number of indoor off-exhibit areas (F_1,24_ = 14.911, P = 0.001) and the number of days trained per month (F_1,24_ = 6.799, P = 0.015) predicted lower baseline FGM concentrations. There were no effects from possible confounding variables (transferred/stationary: F_1,24_ = 0.458, P = 0.505; age: F_1,24_ = 0.154, P = 0.698; how busy was the holding area: F_2,24_ = 0.427, P = 0.657; total space provided F_1,24_ = 0.077, P = 0.784; number of keepers: F_1,22_ = 0.068, P = 0.796; time with access outside: F_2,19_ = 0.321, P = 0.729; enrichment days/month: F_1,24_ = 0.001, P = 0.978).

### Longitudinal monitoring of glucocorticoids

**Fig 2:**
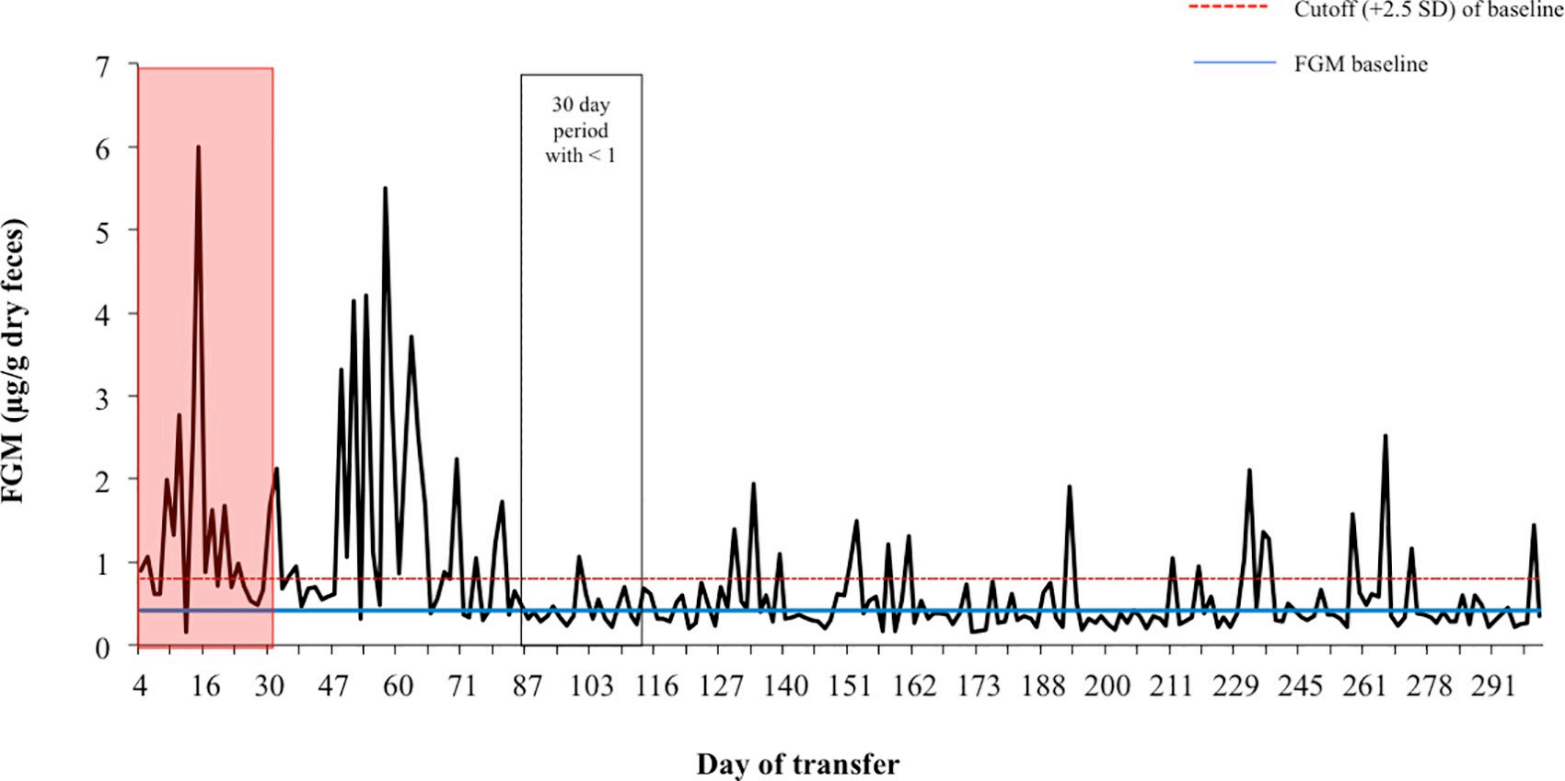
Representative fecal glucocorticoid metabolite profile of a fishing cat (SB# 721) that experienced an institutional transfer. The red shading indicates the time the cat spent in quarantine. The outlined box indicates the return to baseline, defined as 30 consecutive days with ≤ 1 peak above 2.5 standard deviations of the individual’s overall baseline.

**Fig 3:**
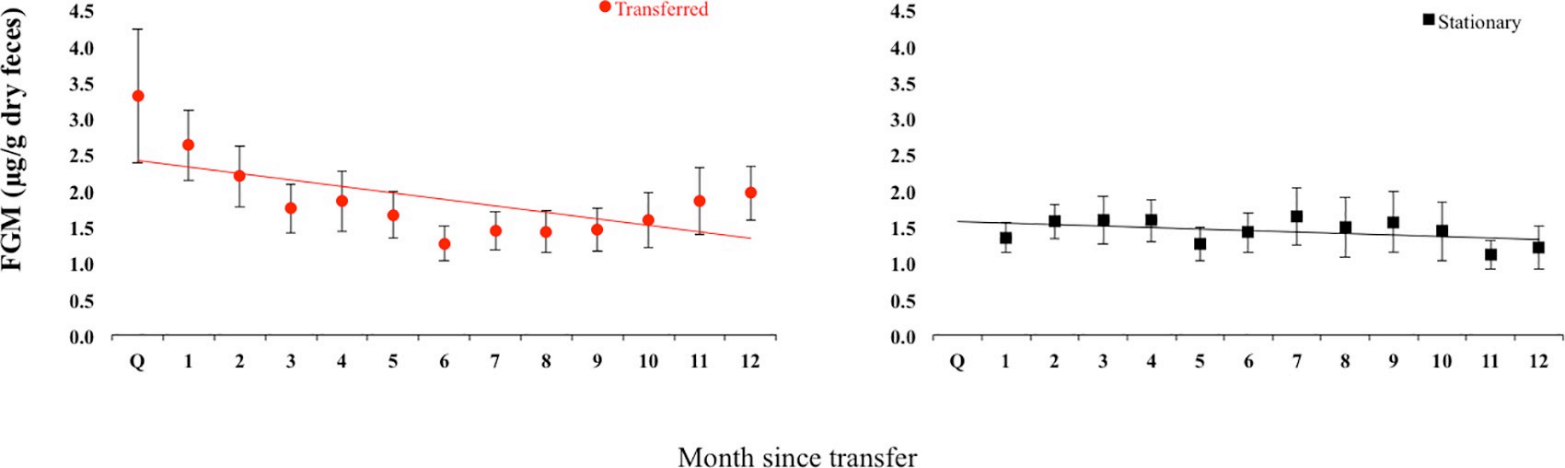
Effect of time on fecal glucocorticoid metabolites (FGM). A negative effect of time on mean FGM concentrations by transfer month was found using a regression analysis on transferred (y = -0.0897x + 2.5003; R^2^ = 0.38; P = 0.003), but not stationary cats (y = -0.0206x + 1.5868; R^2^ = 0.19; = 0.299). Particular note should be taken to the Quarantine period and the first 2 months post release from quarantine for the cats that were transferred, which corresponds to the mean time period calculated for fishing cats to return to baseline after institutional transfer.

Longitudinal monitoring of fishing cats ranged from 6 months to 3 years, depending on the individual. FGM measures were obtained for 26 fishing cats (13 male; 13 female) during 20 institutional transfers (13 male transfers; 7 female transfers) between 2010 and 2013. Post-release FGM concentrations averaged 1.31 ± 0.22 μg/g, and ranged from 0.47–5.28 μg/g. The return to baseline post quarantine release occurred over a mean of 54.37 ± 16.51 days, but ranged from 1–277 days, depending on the individual (Fig 2). When examining the effect of time on mean monthly cortisol levels post-transfer, a significant negative effect was found in the cats that were transferred (r = 0.39, P = 0.003), but not in the stationary (non-transferred) cats (r = 0.19, P = 0.299) (Fig 3).

A significant effect of transfer status was found on FGM concentrations (GLMM: F_1,6712_ = 20.58, P = <0.001), with mean values for transferred individuals being 1.92 ± 0.04 μg/g versus 1.47 ± 0.03 μg/g for stationary individuals. There was, however, no significant effect of the confounding variables sex: F_1,6712_ = 1.055, P = 0.314; age: F_1,6712_ = 3.168, P = 0.075; or rearing: F_1,6712_ = 0.089, P = 0.768 on FGM concentrations. Individuals who were transferred exhibited higher FGM concentrations regardless of sex, age or rearing.

### Chronic illness

**Fig 4:**
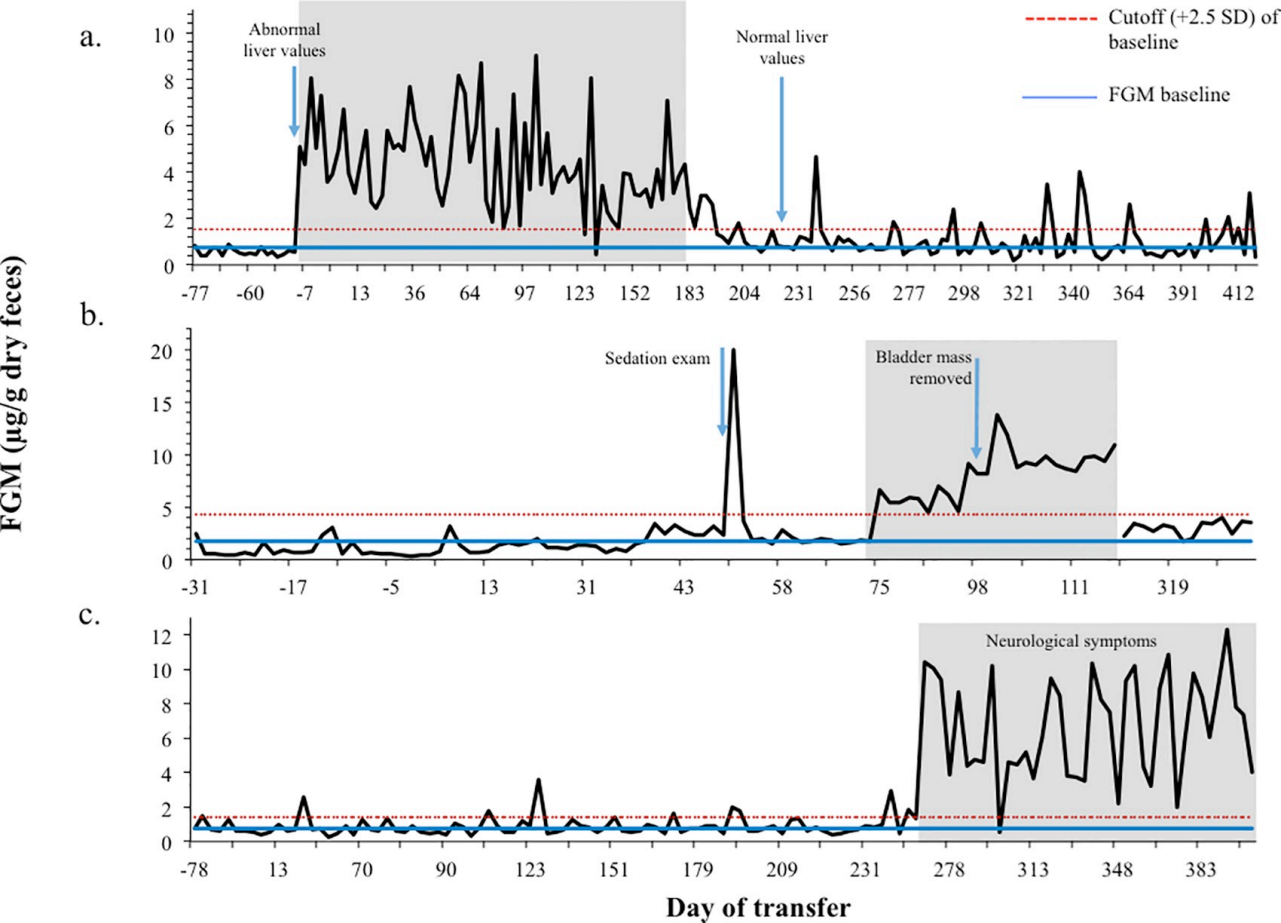
Elevations in fecal glucocorticoid metabolite (FGM) due to chronic illness. Sustained periods of elevation in FGMconcentrations were observed during chronic illness in three fishing cats: (a) SB# 780 presented with elevated liver values during his exam for pre-shipment and was later diagnosed and treated for hepatitis with normal liver values reported 212 days post transfer; (b) SB# 657 treated for a bladder mass tumor, and although collection ended while values were still elevated, baseline values were confirmed several months later when collection resumed (days 313–330); and (c) SB# 440 presented with neurological symptoms and FGMs remained elevated until death.

Three fishing cats diagnosed with a chronic illness exhibited increased FGM during that time (P < 0.05) (Fig 4). SB# 780 presented with elevated liver values during his exam for pre-shipment; he was later diagnosed and treated for hepatitis and normal liver values were reported 212 days post transfer. Baseline FGM values increased from 0.75 to 4.33 μg/g. SB# 657 was treated for a bladder mass tumor. Although collection ended while values were still elevated, baseline values were confirmed several months later when collection resumed (days 313–330). Baseline FGM values increased from 1.72 to 8.19 μg/g. SB# 440 presented with neurological symptoms and FGM increased from 0.73 to 6.69 μg/g, and remained elevated until his passing. FGM in both SB# 657 and 780 returned to baseline after treatment, indicating resolution of the illness, and presumed endogenous stressor.

### Exogenous stressors and social housing

**Fig 5:**
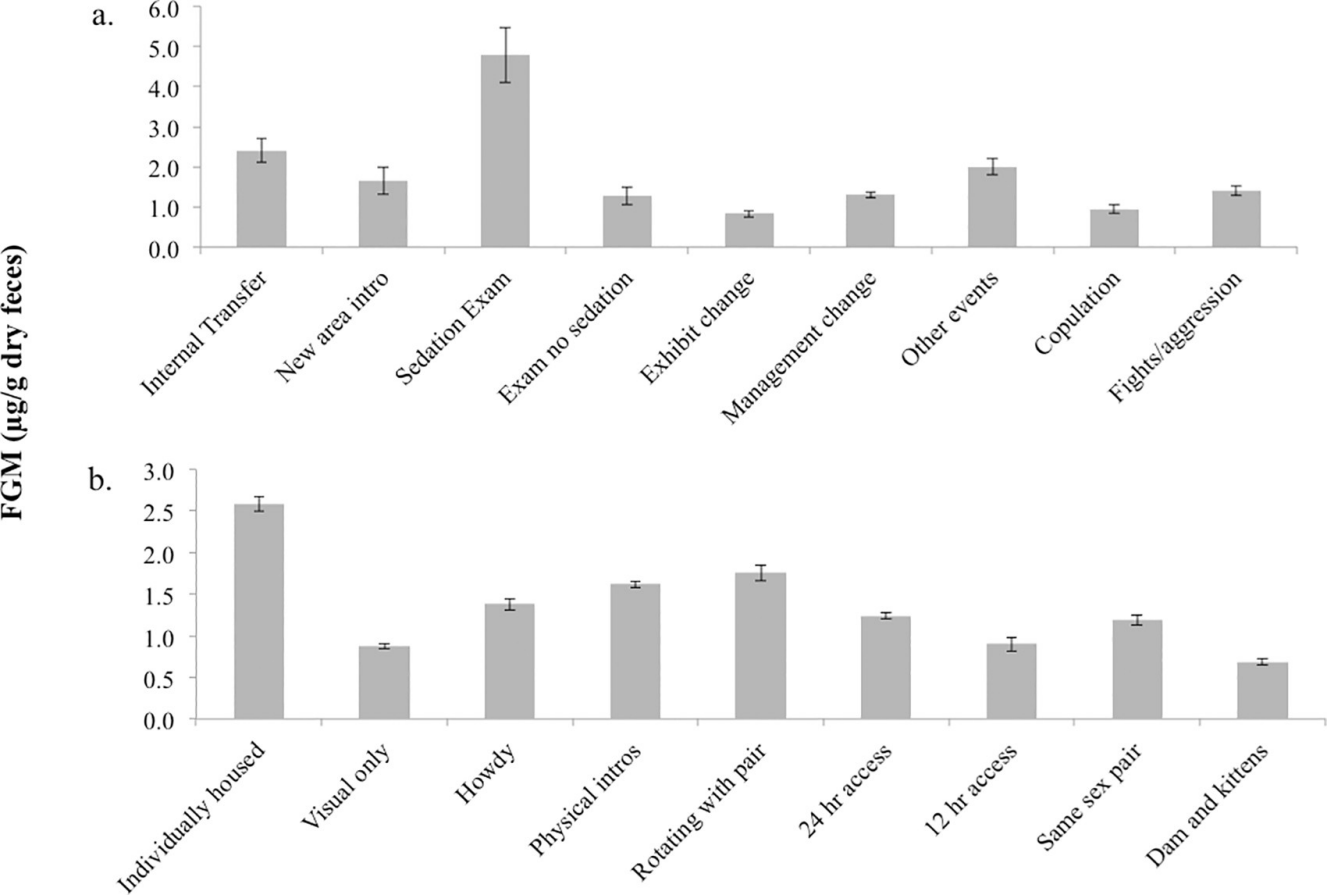
Exogenous stressors and social housing. Effects of exogenous stressors (a) and social housing factors (b) on mean (±SE) fecal glucocorticoid metabolite (FGM) concentrations in fishing cats. Samples were collected from 26 fishing cats housed at AZA institutions in North America between 2010–2013.

Potential exogenous stressors categorized as Events (GLMM: F_8,908_ = 6.68, P = <0.001) were found to have a significant effect on FGM concentrations with no significant effects from possible confounding variables (sex: F_1,908_ = 0.762, P = 0.391; age: F_1,908_ = 2.956, P = 0.088; rearing: F_1,908_ = 0.020, P = 0.888; nor “transfer”: F_1,908_ = 2.04, P = 0.154). Chemical immobilizations (sedation exams) led to the highest mean FGM concentrations, followed by internal transfers and other events, which included construction and facility events where institutions were open after hours (Fig 5a). Social Housing (GLMM: F_8,6712_ = 13.986, P = <0.001) also had an effect on FGM concentrations, with no effects from confounding variables (sex: F_1,6712_ = 1.040, P = 0.317; age: F_1,6712_ = 2.931, P = 0.087; rearing: F_1,6712_ = 0.183, P = 0.672; transfer: F_1,6712_ = 0.482, P = 0.487). Individually housed cats (i.e., solitary animals) had the highest mean FGM concentrations. Individuals undergoing breeding introductions, including howdy, physical introductions and rotating exhibits with their intended mates, also had high mean FGM levels (Fig 5b).

### Predicting breeding success

**Fig 6:**
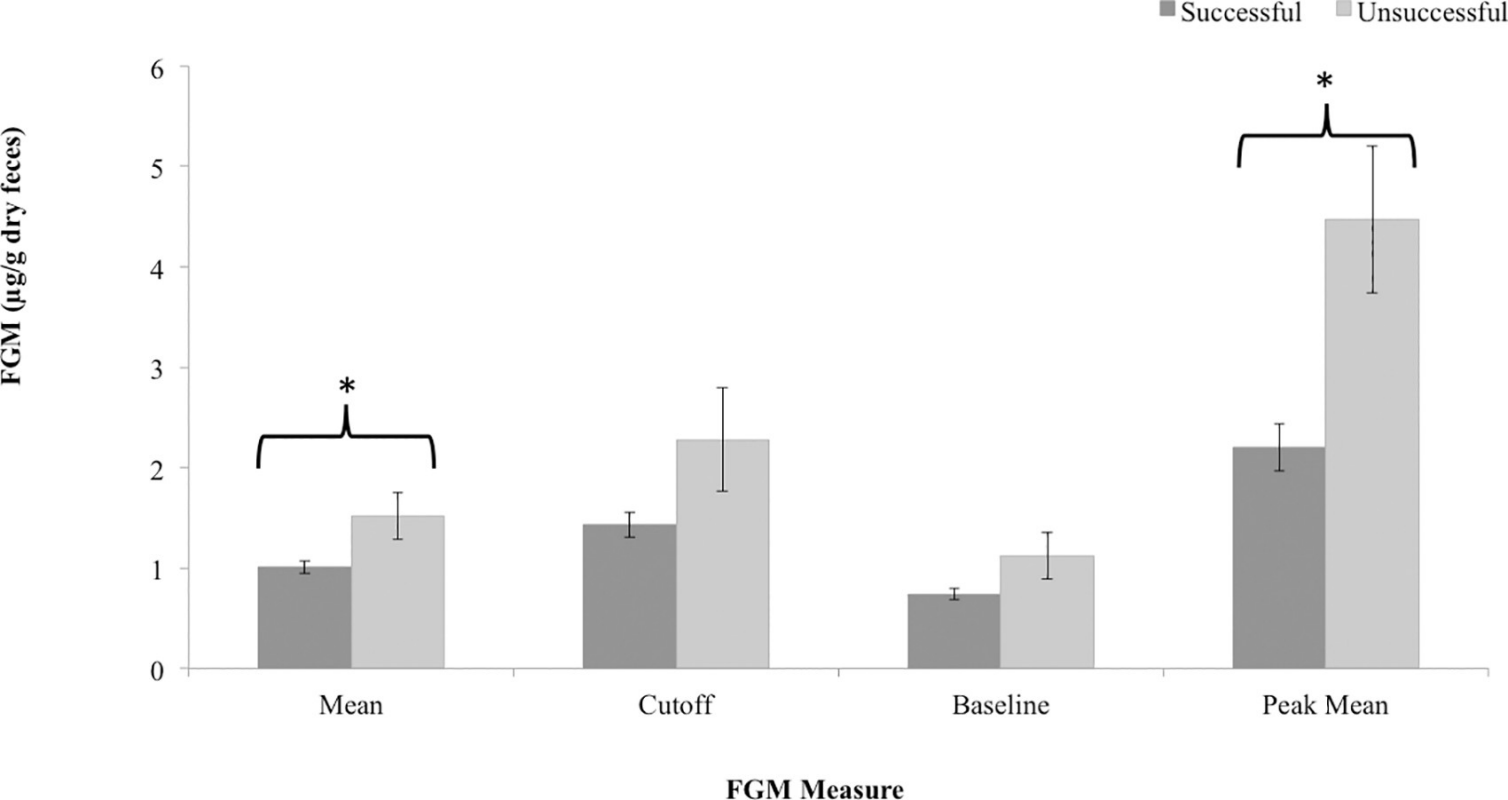
Mean (±SE) fecal glucocorticoid metabolite (FGM) concentrations are presented for fishing cats with observed copulations (successful) and those with no observed copulations (unsuccessful). Results of binomial GLMM revealed that both Mean and Peak Mean FGM measures could predict breeding success in fishing cats.

Of the five FGM measures, both mean and peak mean FGM concentration were significant predictors of breeding success in fishing cats with no effects of the confounding variables transfers, age or rearing. The measures of baseline, cutoff and proportion of peaks were not predictive of breeding success (Table 4). All measures other than proportion of peaks showed higher FGM concentrations among unsuccessful fishing cats (Fig 6) indicating lower FGM concentrations may be indicative of successful breeders.

**Table 4:** GLMM results. Five fecal glucocorticoid metabolite (FGM) measures were calculated in fishing cats (n = 24) during institutional transfers and breeding introductions that took place between 2010–2013. Mean and Peak mean FGM were both significant predictors of individuals exhibiting successful copulations.

Dependent variable	Effect	df	Wald X2	P-value
Mean FGM concentration	transfer	1	0.097	0.755
	age	1	2.476	0.116
	rear	1	1.002	0.317
	**mean**	**1**	**4.521**	**0.033**
Cutoff (2.5 SD of mean FGM concentration)	transfer	1	0.316	0.574
	age	1	1.982	0.159
	rear	1	0.935	0.334
	sd2.5	1	2.055	0.152
Baseline	transfer	1	0.266	0.606
	age	1	1.95	0.163
	rear	1	1.041	0.308
	base_mean	1	2.515	0.113
Peak mean	transfer	1	0.026	0.871
	age	1	1.9	0.168
	rear	1	1.105	0.293
	**peak_mean**	**1**	**6.598**	**0.01**
Proportion of peaks	transfer	1	0.078	0.781
	age	1	0.747	0.387
	rear	1	0.736	0.391
	pop	1	1.892	0.169

Bold typing indicates statistically significant effects. In each model, breeding success was the dependent variable and the predictor was changed to a different cortisol measure with transfer, age and rearing as confounding variables.

## Discussion

Extreme variability in FGM concentrations, regardless of environmental and management conditions, was evident during the longitudinal monitoring of this species. Peaks often occurred even when no potential stressors were recorded, which may have represented stressful events that were missed by animal care staff or natural fluctuations in glucocorticoids for this species. In addition, it was often difficult to pinpoint peaks due to missed samples, even when stressful events were reported. However, when samples were collected within 24–48 hours, various increases ranging from 2–20 times mean baseline were often observed due to exogenous stressors. Institutional transfers were primarily of male fishing cats (13 of 20) and on average it took 2–3 months for FGM concentrations to return to baseline, although there was significant variability depending on the individual. No significant effects of age, method of rearing (hand vs. mother) or sex were found on FGM concentration in this species.

Immunoassay validation is an important first step before using non-invasive fecal glucocorticoid monitoring as a method to assess physiological stress. Inter- and intra- species variation in stress responses is of particular methodological concern [16]. In this study, all four assays detected a significant peak following ACTH. The rise observed in the cortisol EIA reflected a 3.4 times increase in concentration, which is similar to that which has been reported in cheetah [17], clouded leopard [9] and lynx [28]. The cortisol EIA (R4806) was subsequently chosen, and has been shown to be effective in measuring exogenous stressors in other felid species as well [9]. Extreme variability in response to exogenous gonadotropins has been seen in reproductive studies of felids; for example smaller felids such as the ocelot requiring almost double the dose of the much larger cheetah [29]. Results here follow this model with a high dose (80IU/kg) of exogenous ACTH required to stimulate the adrenal gland in the fishing cat. Female ISB 633, that participated in the ACTH challenge had baseline FGM from her three years of monitoring of 0.504 μg/g dry feces, an overall peak mean of 1.61 μg/g dry feces, so it was clear that an elevation to 7.14 μg/g dry feces after the injection of exogenous ACTH indicated a rise due to stimulation of the adrenal gland. Only n = 5 samples during the entire monitoring period rose above 4 μg/g (range = 4.52–5.42 μg/g dry feces). It should be reiterated that it is necessary to have longitudinal monitoring in order to assess each individual’s reaction to potentially stressful events. Without months or even years of data to determine appropriate baseline values it is extremely difficult to pinpoint when a significant elevation, due to a stressor actually occurs.

Immunoreactivity detected by HPLC suggests that the cortisol antibody detected primarily nonenzyme-hydryolyzable, water soluble fractions, and not free cortisol in this species. These results are similar to those obtained previously in the domestic cat injected with ^3^H-cortisol [10] and measured with a cortisol RIA. In this study, because these polar metabolites constituted the primary immunoreactivity, and the ACTH challenge clearly represented an increase (344%) in FGM, that cortisol antibody may be cross-reacting with other, as yet unidentified, glucocorticoid metabolites.

Chronic illness resulted in sustained elevations in adrenal activity in all three individuals diagnosed with long-term illness during the study period; increasing baseline levels were observed to be four to nine times higher than those in the same individual when healthy. Disease can affect the HPA axis [30], specifically during inflammatory illness [31]; therefore, the consistently high concentrations of FGM detected during chronic illness may be indicative of such a physiological response.

Chemical immobilizations (examinations under sedation) resulted in the highest fecal FGM concentrations, followed by permanent internal transfers, which typically mimicked an inter-institutional transfer in that staffing, exhibit and management were all changed. The “other events” category was the next highest cause for increased FGM concentrations, which included construction that several cats experienced during the study period (n = 5). Exposure to construction has been shown to decrease physical activity and increase FGM in several other felid species [32]. The “other events” category also included after hours events. Such events have been shown to affect reproductive cyclicity in seasonal species, especially if natural light conditions are altered [33], and these can be stressful presumably due to disturbances that occur during periods that are typically quiet [34].

Social housing also was found to have a significant effect on FGM levels in fishing cats. Observations of wild fishing cats are scarce and the ecology of this species is still mostly unknown, although field research has increased in recent years [35–39]. Most camera trap photos reveal single individuals or females with offspring [36,40], leading researchers to believe they are largely solitary in the wild. However, in zoos, when fishing cats were housed alone, FGM concentrations were the highest, with a mean value that was almost double the post-release level found in the species. Even cats provided visual access to another fishing cat had lower FGM concentrations than singletons.

Most felid species are considered primarily solitary; however, in captivity many of these species can adapt to “unnatural” social groupings of same sex or breeding pairs for long periods of time [22]. One extreme example is the clouded leopard, where individuals are introduced as breeding pairs when they are juveniles and can then maintain a strong pair-bond for life [41]. Established fishing cat breeding pairs, same sex pairs, and dams with kittens, all exhibited mean FGM concentrations below the overall mean average for the species. These results indicate that positive social interactions may help reduce FGM levels (and presumably stress) in this species. It should be noted that during this study period, the only same-sex pairs were males. Therefore, it should not be assumed that female same-sex pairs would respond similarly. In fact it has been found in cheetahs that socially housing females can suppress ovarian cyclicity [4], so the impact of housing female fishing cats socially should be investigated in the future.

Higher FGM concentrations were also recorded in breeding pairs that were being established, specifically, during howdy (mesh to mesh access without physical contact) and physical introductions and when un-established pairs were being rotated through shared exhibits. Rotation through different holding and exhibit areas is commonly performed when breeding introductions of new individuals begin, especially if there is only one large exhibit space available. Because introductions to new individuals, especially for breeding, are considered stressful events for most animals [42] these findings were expected.

Fishing cats that were trained more frequently in this study had lower FGM concentrations. Training or operant conditioning can be viewed as one way to enhance and strengthen the keeper-animal relationship [43]. A positive relationship can be essential to the captive management of a species. Increased time spent with an animal keeper can positively affect reproductive success [14] and reduce glucocorticoid levels [6] in felids. Besides building and maintaining a positive relationship between animal and keeper, training can reduce stress by giving the animal more control over their environment and care, which can help to reduce adrenal activity especially during potentially stressful events [44]. Training prior to the transfer of tigers (*Panthera tigris*) lowered glucocorticoid levels [45]. Moreover, chimpanzees (*Pan troglodytes*) trained for voluntary sedation procedures showed significantly lower levels of several physiological measures associated with the stress response, such as glucose and white blood counts [46].

Access to indoor off-exhibit areas predicted lower FGM concentrations, indicating that providing private areas (2–4 enclosures) where cats can take refuge from the public in a private, quiet, undisturbed area may lower stress. In captivity, exposure to the public has been shown to increase glucocorticoid levels in several species [47]. In a study of cheetah transfers, movement of individuals from an off-exhibit to an on-exhibit area resulted in an increase in their FGM baseline; generally cheetahs housed on-exhibit have higher baselines overall [27]. Many small cats are prey species for other larger carnivores [48,49]. Therefore, hiding spaces are important for providing escape areas that can help them feel secure in their captive environment [22,14]. These indoor off-exhibit areas may provide the security that fishing cats need in captivity.

Finally, breeding success in this species was predicted by lower overall mean and peak mean FGM concentrations. Therefore, instituting management decisions, such as positive animal keeper interactions through training, providing multiple indoor off-exhibit refuge areas, and social housing of established breeding or same sex pairs, may all contribute to better welfare and greater captive breeding success in the fishing cat via reducing stress levels.

## Supporting information

S1 Table Pearson’s correlation of management variables. Results from institutions (n = 16) holding fishing cats (*Prionailurus viverrinus*) in North American zoos. Significant results are bold. * Correlation is significant at the p < 0.05 level (two-tailed) ** Correlation is significant at the p < 0.01 level (two-tailed).
(PDF)

S1 Fig High performance liquid chromatography (HPLC). Separation during HPLC of metabolized cortisol metabolites in fishing cat feces. Immunoreactivity of each fraction was determined by cortisol EIA (R4806).
(TIF)
