# Supplementary material for: Longitudinal fecal hormone monitoring of adrenocortical function in zoo housed fishing cats (Prionailurus viverrinus) during institutional transfers and breeding introductions
Source: PLoS One. 2020 Mar 18;15(3):e0230239. doi: 10.1371/journal.pone.0230239 (PMC7080239; doi:10.1371/journal.pone.0230239)
Supplement: S1 Table — Results from institutions (n = 16) holding fishing cats (Prionailurus viverrinus) in North American zoos. Significant results are bold. * Correlation is significant at the p < 0.05 level (two-tailed) ** Correlation is significant at the p < 0.01 level (two-tailed). (PDF) [file pone.0230239.s001.pdf]

Management variables		# indoor off exhibit	total indoor enclosure size	indoor enclosure height	# outdoor on exhibit enclosures	# outdoor off exhibit enclosures	total outdoor enclosure size	outdoor enclosure height	how busy is holding	# keepers	average years experience	previous exp.	free contact	fast days	amount of time given access outside	visual access to carnivores	# training	# enrichment	# of nestboxes
# indoor on exhibit enclosures	Pearson Correlation	0.21	0.29	0.45	-0.35	0.48	-0.24	-0.24	**.837****	0.19	0.26	-0.28	**.630****	-0.01	-0.06	0.00	-0.06	-0.14	**.744***
	Sig. (2-tailed)	0.43	0.32	0.16	0.19	0.06	0.60	0.61	**0.00**	0.47	0.32	0.41	**0.01**	0.97	0.81	1.00	0.83	0.60	**0.01**
# indoor off exhibit enclosure	Pearson Correlation		0.29	-0.03	-0.21	0.29	-0.21	0.06	0.32	0.25	0.13	0.41	-0.01	-0.28	0.23	-0.14	0.23	0.41	0.02
	Sig. (2-tailed)		0.32	0.93	0.45	0.28	0.66	0.90	0.22	0.35	0.64	0.22	0.97	0.30	0.39	0.60	0.39	0.11	0.95
total indoor enclosure size	Pearson Correlation			**.728***	**-.724****	-0.05	-0.10	-0.11	0.14	0.41	0.52	-0.39	-0.12	-0.09	0.49	-0.15	0.06	0.39	-0.20
	Sig. (2-tailed)			**0.01**	**0.00**	0.88	0.83	0.82	0.65	0.15	0.06	0.27	0.69	0.76	0.08	0.61	0.84	0.17	0.62
indoor enclosure height	Pearson Correlation				**-.624***	0.15	-0.27	-0.20	0.24	0.38	0.19	-0.42	0.10	-0.06	0.21	0.14	-0.12	0.01	0.04
	Sig. (2-tailed)				**0.04**	0.67	0.61	0.68	0.48	0.26	0.58	0.30	0.78	0.85	0.53	0.69	0.73	0.99	0.93

Management variables		# indoor off exhibit	total indoor enclosure size	indoor enclosure height	# outdoor on exhibit enclosures	# outdoor off exhibit enclosures	total outdoor enclosure size	outdoor enclosure height	how busy is holding	# keepers	average years experience	previous exp.	free contact	fast days	amount of time given access outside	visual access to carnivores	# training	# enrichment	# of nestboxes
# outdoor on exhibit enclosures	Pearson Correlation					0.24	0.01	-0.19	-0.22	-0.49	-0.42	0.35	-0.08	0.10	**-.797****	0.12	0.39	-0.14	0.32
	Sig. (2-tailed)					0.37	0.99	0.68	0.41	0.05	0.11	0.30	0.77	0.72	**0.00**	0.66	0.13	0.60	0.36
# outdoor off exhibit enclosures	Pearson Correlation						0.04	-0.04	.497*	-0.22	0.28	-0.04	0.44	0.00	-0.38	-0.33	0.31	0.03	**.813****
	Sig. (2-tailed)						0.93	0.93	0.05	0.41	0.29	0.91	0.09	1.00	0.15	0.21	0.25	0.92	**0.00**
total outdoor enclosure size	Pearson Correlation							-0.11	-0.32	-0.38	-0.21	-0.28	-0.11	-0.27	0.14	-0.20	-0.31	0.30	-0.10
	Sig. (2-tailed)							0.84	0.48	0.40	0.65	0.65	0.82	0.56	0.77	0.67	0.49	0.52	0.87
outdoor enclosure height	Pearson Correlation								-0.43	-0.56	-0.15	0.42	-0.13	0.31	**.861***	-0.38	-0.09	0.50	0.00
	Sig. (2-tailed)								0.34	0.19	0.75	0.48	0.78	0.50	**0.01**	0.41	0.85	0.26	1.00
how busy is holding	Pearson Correlation									0.46	0.39	-0.29	**.750****	-0.23	-0.10	-0.30	0.15	-0.21	**.720***
	Sig. (2-tailed)									0.07	0.14	0.38	**0.00**	0.39	0.70	0.27	0.58	0.45	**0.02**

Management variables		# indoor off exhibit	total indoor enclosure size	indoor enclosure height	# outdoor on exhibit enclosures	# outdoor off exhibit enclosures	total outdoor enclosure size	outdoor enclosure height	how busy is holding	# keepers	average years experience	previous exp.	free contact	fast days	amount of time given access outside	visual access to carnivores	# training	# enrichment	# of nestboxes
# keepers	Pearson Correlation										0.27	-0.04	0.24	-0.16	0.43	-0.40	0.08	0.19	-0.16
	Sig. (2-tailed)										0.32	0.90	0.36	0.55	0.09	0.12	0.77	0.49	0.66
average years experience	Pearson Correlation											-0.56	0.29	-0.02	0.24	**-.529***	0.01	0.12	0.16
	Sig. (2-tailed)											0.08	0.28	0.93	0.36	**0.04**	0.98	0.67	0.67
free contact	Pearson Correlation													0.13	-0.10	-0.22	-0.12	-0.44	**.633***
	Sig. (2-tailed)													0.62	0.71	0.42	0.65	0.09	**0.05**
fast days	Pearson Correlation														-0.08	0.20	-0.31	0.06	-0.16
	Sig. (2-tailed)														0.78	0.45	0.24	0.82	0.67
amount of time given access outside	Pearson Correlation															-0.33	-0.27	0.41	-0.56
	Sig. (2-tailed)															0.21	0.32	0.12	0.09
visual access to carnivores	Pearson Correlation																-0.36	-0.47	-0.29
	Sig. (2-tailed)																0.17	0.07	0.41

Management variables		# indoor off exhibit	total indoor enclosure size	indoor enclosure height	# outdoor on exhibit enclosures	# outdoor off exhibit enclosures	total outdoor enclosure size	outdoor enclosure height	how busy is holding	# keepers	average years experience	previous exp.	free contact	fast days	amount of time given access outside	visual access to carnivores	# training	# enrichment	# of nestboxes
# training days/month	Pearson Correlation																	0.30	0.32
	Sig. (2-tailed)																	0.26	0.37
# enrichment days/month	Pearson Correlation																		-0.16
	Sig. (2-tailed)																		0.66
