# Supplementary figures and images for: Longitudinal fecal hormone monitoring of adrenocortical function in zoo housed fishing cats (Prionailurus viverrinus) during institutional transfers and breeding introductions
Source: PLoS One. 2020 Mar 18;15(3):e0230239. doi: 10.1371/journal.pone.0230239 (PMC7080239; doi:10.1371/journal.pone.0230239)

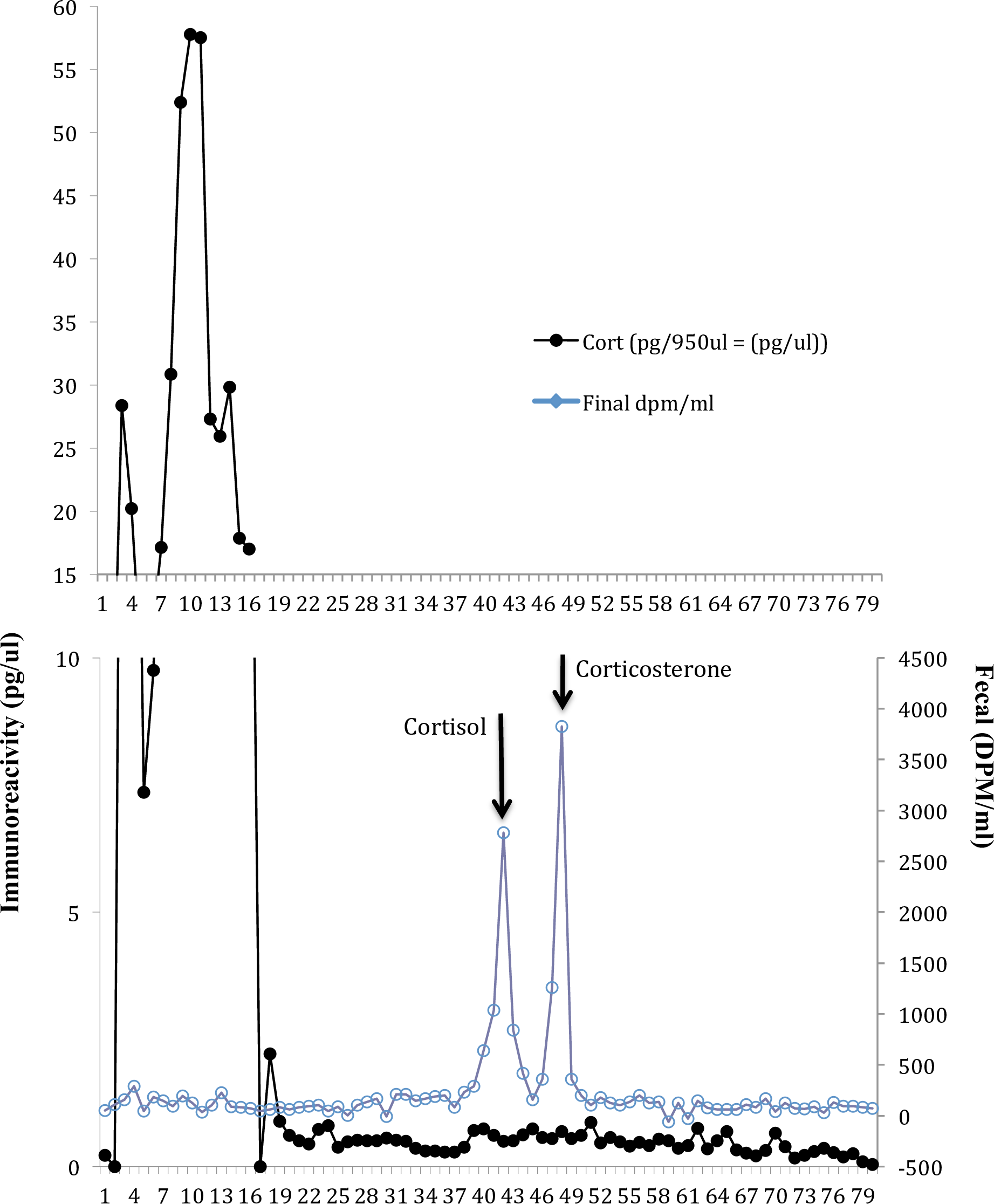

Supplement: S1 Fig — Separation during HPLC of metabolized cortisol metabolites in fishing cat feces. Immunoreactivity of each fraction was determined by cortisol EIA (R4806). (TIF) [file pone.0230239.s002.tif]
